# TMD and chronic pain: A current view

**DOI:** 10.1590/2176-9451.20.1.127-133.sar

**Published:** 2015

**Authors:** Bruno D'Aurea Furquim, Lívia Maria Sales Pinto Flamengui, Paulo César Rodrigues Conti

**Affiliations:** 1 PhD in Oral Rehabilitation, University of São Paulo (USP); 2 PhD in Oral Rehabilitation, School of Dentistry - USP/Bauru; 3 Full professor, Department of Prosthesis, School of Dentistry - USP/Bauru

**Keywords:** Temporomandibular disorder, Myofascial painful syndrome, Central nervous system sensitization, Autonomic nervous system

## Abstract

This review aims at presenting a current view on the physiopathologic mechanisms associated with temporomandibular disorders (TMDs). While joint pain is characterized by a well-defined inflammatory process mediated by tumor necrosis factor-α and interleukin, chronic muscle pain presents with enigmatic physiopathologic mechanisms, being considered a functional pain syndrome similar to fibromyalgia, irritable bowel syndrome, interstitial cystitis and chronic fatigue syndrome. Central sensitization is the common factor unifying these conditions, and may be influenced by the autonomic nervous system and genetic polymorphisms. Thus, TMDs symptoms should be understood as a complex response which might get worse or improve depending on an individual's adaptation.

## Abstract (pt)

Esta revisão teve como objetivo apresentar uma visão atualizada dos mecanismos fisiopatológicos relacionados às disfunções temporomandibulares (DTMs). Enquanto a dor articular é caracterizada por um processo inflamatório bem descrito - mediado pelo fator de necrose tumoral alfa (TNF-α) e interleucinas -, a dor muscular crônica apresenta mecanismos fisiopatológicos mais obscuros, sendo considerada uma síndrome dolorosa funcional, assim como a fibromialgia, a síndrome do intestino irritável, a cistite intersticial e a síndrome da fadiga crônica. A sensibilização central é o processo comum, unificador, dessas condições, podendo sofrer influência do sistema nervoso autonômico e de polimorfismos genéticos. Portanto, os sintomas das DTMs devem ser entendidos como uma resposta complexa, podendo ser amplificados ou atenuados em função da adaptação individual.

## INTRODUCTION

Temporomandibular disorders (TMDs) represent a set of muscle-skeletal disorders associated with the masticatory system and a number of symptoms. Pain is the most common symptom usually concentrated in masticatory muscles and/or temporomandibular joints (TMJs), but exacerbated by mandibular movement and stomatognathic functions.^1 ^TMD seems to be of multifactorial etiology, including parafunctional habits, bruxism, deleterious body posture, occlusal features, growth abnormalities, trauma, overload and stress.^2^^-^ 8

Despite extensive literature on the topic, there is a need for further prospective, controlled, randomized, long-term clinical trials in order to establish a concrete cause and effect relationship. On the other hand, significant advance has been made with regard to the physiopathologic mechanisms associated with this condition. 

## JOINT PAIN AND INFLAMMATORY CYTOKINES

The pathophysiology of TMJ pain is better understood than masticatory muscle pain; thus, the former is a convenient starting point to discuss TMDs pain mechanisms.^9^ Disc displacement and degenerative joint pain are commonly associated with TMJ. Osteoarthritis is characterized by articular cartilage deterioration and abrasion, as well as by remodeling thickening of subjacent bone. It causes secondary inflammatory reactions, such as joint effusion, revealed by magnetic resonance. The TMJ might also be affected by rheumatoid arthritis, an autoimmune condition that results in inflammatory joint destruction. Chemical mediators and cytokines play an important role in both rheumatoid arthritis and osteoarthritis. Regardless of the pathological condition, degenerated TMJ might lead to a number of morphological defects associated with pain and significant loss of articular function.^9^^-^ 13

TMJ inflammation results in the release of various proinflammatory cytokines, particularly tumor necrosis factor-α (TNF-α) and interleukins,^14^^,^ 15 ^,^ 16 which contribute to articular cartilage remodeling and deterioration.^17^ Interleukin-1 (IL-l) and interleukin-6 (IL-6) have been found in cases of osteoarthritis and temporomandibular joints with internal disarrangements.^18^^,^ 19 ^,^ 20

Cytokines are mainly produced by macrophages penetrating into the synovium. Synovium inflammation affects the viscosity of synovial fluid and leads to insufficient lubrication and nourishment of cartilage and disc.^21^

Inflammatory mediators stimulate the nociceptors of TMJ and increase the release of CGRP (calcitonin gene related peptide) and substance P, which result in swelling, redness and a rise in temperature. This process is known as neurogenic inflammation.^22^ Increased nociceptive stimuli in inflamed joints also contribute to inducing central sensitization and reflex of mandibular muscles (protective contraction).^15^^,^ 23

## TMD: A FUNCTIONAL PAIN SYNDROME

Chronic TMD, especially myofascial TMD, is considered a functional pain syndrome similar to fibromyalgia, irritable bowel syndrome, interstitial cystitis and chronic fatigue syndrome. These conditions appear to have common etiological factors which explain the great comorbidity of symptoms. It is interesting to notice that functional disorders tend not only to cumulatively affect an individual, but also to present central sensitization and amplified pain perception. Such disorders have similar treatment response, and may be treated with antidepressant drugs and cognitive behavioral therapy. The pathophysiologic mechanisms of such pain conditions remain unknown. However, it is believed that amplified pain perception, alterations in brain activity as well as in immune and neuroendocrine activities, and genetic predisposition may be involved. Further studies should be capable of revealing a predominant or unifying mechanism that explains such functional alterations.^24^^,^ 25 ^,^ 26

In cases of functional pain syndrome, pain is no longer a protection factor. Pain is spontaneously felt and might be triggered by innocuous stimuli (allodynia); it might be excessive and prolonged and occurring in response to nociceptive stimuli (hyperalgesia); and it might spread beyond the injured site (secondary hyperalgesia).^27^ Should hyperalgesia occur after tissue is injured, it results from increased sensitivity of primary afferent nociceptors found around the injured site (peripheral sensitization)^28^and increased excitability of secondary afferent nociceptors found in the spinal cord (central sensitization).^29^

## PAIN CHRONICITY

The phenomenon of peripheral sensitization occurs as a result of inflammatory response provoked by a tissue injury. Should that be the case, allodynia and hyperalgesia occur due to inflammatory mediators released at the site of lesion. For instance, whenever a tooth is extracted, the site of inflammation presents with increased sensitivity to pressure (hyperalgesia) mediated by sensitized nociceptors. Nevertheless, such reaction is expected to restore within a reasonable period of time due to decreased nociceptors activity and consequent decreased afferent activity of the dorsal horn. However, the inflammatory process and its consequent afferent activity might be intense enough so as to establish a central process.^30^

C-fibers are the first nociceptors involved in central sensitization onset, as they produce slow synaptic currents and repetitive stimuli, thereby increasing depolarization in the spinal cord dorsal horn as a result of activating the calcium channels that depend on binders. Initially, calcium channels are opened, quickly and for a short period of time, by AMPA (α-Amino-3-hydroxy-5-methyl-4-isoxazolepropionic acid) receptors. This process allows calcium ions to penetrate the cell and favors depolarization of wide dynamic range neurons which can respond to a large variety of stimuli. In addition to activating ionotropic receptors, glutamate and substance P also activate metabotropic receptors, thereby releasing more calcium to intracell vesicles, increasing the concentration of calcium ion and, as a result, activating protein-kinase enzymes that phosphorylate the N-methyl-D-aspartate (NMDA) receptor. In normal conditions, the channel bound to the NMDA receptor is blocked by magnesium ions. Once this receptor is activated, it is phosphorylated and magnesium ions are released, thereby opening the channel and allowing calcium ions to enter the cell. Unlike what happens with the AMPA receptor, activation of the NMDA receptor is enduring and hardly ever reverted.^30^^,^ 31 ^,^ 32

Although activation of NMDA receptors seem to play a major role in central sensitization, a single molecular mechanism responsible for the process has not yet been identified, as it might be mediated by different processes capable of producing a variety of alterations in the somatosensory system. Of the many alterations, the following apply: increased excitability of neuronal membrane, facilitation of synapses and decreased inhibitory influence of dorsal horn neurons. Thus, central sensitization might lead to pain despite absence of pathologies or peripheral pain stimuli and, therefore, it should target the central nervous system, not the peripheral one.^27^^,^ 33

## EMOTIONS AND THE AUTONOMIC NERVOUS SYSTEM

Lorduy et al^24^ found that central sensitization symptoms are associated with stronger emotional suffering in TMD patients. Other studies reveal that psychological factors vary among TMD patients and control groups.^34^^,^ 35 ^,^ 36 Additionally, TMD patients suffering from depression and anxiety have an increased risk of feeling joint and muscle pain, respectively.^37^

The relationship established between anxiety or stress and TMD is simply explained by the greater contraction of masticatory muscles happening as a result of TMD, since muscle hyperactivity is one of the most frequent mechanisms influencing myofascial pain.^38^^,^ 39 An experiment in which patients were subject to stressful conditions revealed that myofascial TMD patients presented with increased electromyographic activity of masseter and frontal muscles in comparison to the control.^40^^,^ 41 Nevertheless, there is a more complex explanation for such relationship. 

Mild negative emotions might favor the occurrence of pain. In this context, whenever damage is unpredictable, pain plays an important role in detecting risky situations so as to preserve tissue integrity. It is an adaptive means of promoting environmental monitoring, a sensory monitoring mechanism to increase threat detection.^42^^,^ 43 ^,^ 44 Having the expectation to feel pain may increase pain sensitivity, particularly when the moment of pain cannot be anticipated.^44^

In situations involving intense negative emotions, whenever thread is imminent and predictable, fighting and escapement reactions establish hypoalgesia as a defense mechanism. An interesting experiment revealed that Vietnam veterans with post-traumatic stress disorder (PTSD) reported 30% less pain when stimulated by heat after being exposed to battle videos. There was no reduction in pain intensity when veterans were subject to naloxone, an opioid receptor antagonist. Results clearly revealed analgesia induction mediated by opioid and induced by stress in PTSD patients.^45^

The amygdala detects danger by causing fear and anxiety and, as a result, putting us in state of alert. The connections between amygdala and periaqueductal gray are involved in the modulation of emotion-mediated nociception. An unregulated hazard detection circuit in functional pain syndrome patients might decrease the threshold of negative, intense, long-term emotional experiences. Thus, unlike healthy patients, intense negative emotions might lead to hyperalgesia, not hypoalgesia.^46^^,^ 47

Diffuse noxious inhibitory controls (DNIC) are the main endogenous pain inhibitory systems. The literature asserts that a nociceptive stimulus suppresses another nociceptive stimulus ("pain inhibits pain" mechanism) provided that body surfaces under stimulation are at a certain distance. Patients with fibromyalgia subjected to cold pressor tests are less likely to respond to DNIC, thereby making endogenous pain inhibitory system deficiency explicit.^48^^,^ 49

Robinson et al^50^ proved that anxiety is positively associated with the process of temporal somatization, thereby suggesting that anxiety might contribute to central pain processing. The same effect has been repeated by Granot et al.^51^ Likewise, Edwards et al^52 ^suggested that pain catastrophizing might produce the same effect. Not coincidentally, a number of studies highlight the correlation between negative emotions and painful functional disorders.^53^

Psychological stress is known for inducing adaptive responses of physiological systems, including increased hypothalamo-hypophyseal adrenal system activity. These responses induce cortisol secretion by the adrenal cortex and increase sympathetic adrenal medullary system (SAM) activity which, in turn, isolates adrenalin and noradrenaline through peripheral sympathetic nerve endings and adrenal medulla.^54^^-^ 57 Trait-anxiety (State-Trait Anxiety Inventory - STAI) and altered plasmatic cortisol concentrations, adrenalin and noradrenaline were significantly associated after psychologically-induced stress (mental arithmetic test) in myofascial TMD patients; however, healthy individuals did not behave accordingly. Results suggest that anxiety levels, particularly trait-anxiety, might be associated with greater sensitivity in hypothalamo-hypophyseal adrenal system and sympathetic adrenal medullary system in patients under myofascial pain.^35^

Emerging evidence suggests that unregulated autonomic nervous system contributes to TMD development and chronicity.^58^^-^ 62 When compared to healthy individuals, TMD patients present with autonomic activity dysfunction characterized by decreased heart rate variability at rest as well as in response to physical (standing position) and psychological (Stroop test) stressors, thereby proving that cardiac parasympathetic tone remained low at all times and frequency in comparison to control. TMD patients also present reduced baroreceptor sensitivity.^63^ Other studies also suggest greater sympathetic tone as a result of unregulated central in TMD and other chronic muscle diseases patients.^61^^,^ 64

Chalaye et al^65^ confirm the presence of increasing somatic hyperalgesia levels in irritable bowel syndrome and fibromyalgia patients. Likewise, the authors also found a dysfunctional pattern for pain inhibition followed by abnormal autonomic responses, which kept patients (especially fibromyalgia ones) in a state of sympathetic hyperactivity.^65^ Recent results reveal reduced baroreceptor sensitivity in fibromyalgia patients.^66^^,^ 67 ^,^ 68 Baroreceptor activity has also been associated with descending pain inhibitory system efficiency.^69^^,^ 70 ^,^ 71 Therefore, abnormal baroreceptor activity not only explains why fibromyalgia patients present with deficient descending pain inhibition along with poor anatomical adjustments,^72^ but also why they often suffer from comorbidities such as fatigue, orthostatic intolerance, sleep disorders and impaired cognition.^66^^,^ 73

Unregulated central in TMD patients causes the autonomic nervous system to react less to physical or psychological stress, since the sympathetic tone is high even at rest. This characteristic has been associated with COMT gene variables (SNPs or single-nucleotide polymorphisms)^74^ responsible for provoking an hyperadrenergic state. COMT variables are also associated with hypervigilance, anxiety, pain hypersensitivity and inefficient opioid system.^75^^,^ 76 ^,^ 77 Thus, COMT gene polymorphism illustrates how genetics embraces a vast universe of investigation and research.

The different physiopathologic mechanisms involved in the multifactorial nature of TMDs suggest that distinct genetic loci are connected in such a way that each locus produces minor effects and interacts with environmental exposure.^78^^,^ 79

## CONCLUSION

TMD symptoms should be understood as a complex individual response with unique complaints and which might get worse or improve depending on an individual's genetic composition.

Due to multiple etiological factors and different individual adaptation, multidisciplinary therapy should be encouraged. Likewise, future research elucidating neurobehavioral processes underlining chronic pain should also be encouraged.
